# Supplementary material for: The discovery of novel noncoding RNAs in 50 bacterial genomes
Source: Nucleic Acids Res. 2024 Apr 22;52(9):5152–65. doi: 10.1093/nar/gkae248 (PMC11109978; doi:10.1093/nar/gkae248)
Supplement: gkae248_Supplemental_Files [file gkae248_supplemental_files.zip › Narunsky 50 Genomes RNA Motifs Files.zip!/Publication/61-Geobacter_sp_M21/Figures/LRC-61-21-NC_012918_1_IGR_82_602198_602458.sto.pdf]

LRC-61-21-NC_012918_1_IGR_82_602198_602458.sto.r2r

5′-○○○○○○○○○○○○○○○○○○○○○○○○○○○○○○○○○○○○○○○○○○○○○○○○○○○○○○○YYUU●CC●CGYCYGC●●UYUUGCCYGCAGYUU●C●C○○CUYCY●UCCGAUA●●YYCYGYAG●●GGYA●CGCGCCGC●CC●●●●GACA●A●CYU●CCG●●●Y●GYR●URAAA●○CC●UUUGAAUUGACAACU●YGC●ARAUUCCGCUAUUCUGCGC●○C●GUGCGU●CGC●●○C●GU○○○●●CCCG●AAACAGGUAUUG
